# Emerging waterborne infections in health-care settings.

**DOI:** 10.3201/eid0702.010225

**Published:** 2001

**Authors:** A M Emmerson

**Affiliations:** Queen's Medical Centre, Nottingham, United Kingdom. michael.emmerson@nottingham.ac.uk

## Abstract

Water is used in vast quantities in health-care premises. Many aquatic microorganisms can survive and flourish in water with minimal nutrients and can be transferred to vulnerable hospital patients in direct (e.g., inhalation, ingestion, surface absorption) and indirect ways (e.g., by instruments and utensils). Many outbreaks of infection or pseudoinfection occur through lack of prevention measures and ignorance of the source and transmission of opportunistic pathogens.


### Emerging Waterborne Infections in Health-Care Settings

**Alfred M. Emmerson**
Queen's Medical Centre, Nottingham, United Kingdom

Water is used in vast quantities in health-care premises. Many aquatic microorganisms can survive and flourish in water with minimal nutrients and can be transferred to vulnerable hospital patients in direct (e.g., inhalation, ingestion, surface absorption) and indirect ways (e.g., by instruments and utensils). Many outbreaks of infection or pseudoinfection occur through lack of prevention measures and ignorance of the source and transmission of opportunistic pathogens.

Wholesome (clear, palatable, and safe) drinking water is fundamental to public health. More than 95% of the population of the United Kingdom have a public supply of piped drinking water, almost all chlorinated and some fluorinated. The bacteriologic quality of drinking water has been maintained in accordance with well-established guidelines (1). In the United Kingdom, water providers have been required by law since 1847 to supply wholesome drinking water. However, it is only in the most recent legislation, the Water Act 1989 and its accompanying Water Supply (Water Quality) Regulations (2), that a definition of "wholesome" appears (3). Directives are one of the means by which European Community legislation is applied to member states. Two of these, the Surface Water Directive and the Sampling Directive, concern the use of surface water as a source of drinking water; a third, the Drinking Water Directive (4,5), is intended to ensure a wholesome water supply for drinking and for food and drink manufacture.

### Public Water Companies

Public water companies have considerable expertise and resources to ensure that their supply systems are designed, operated, and monitored to comply with the minimum requirements of the law. U.K. legislation regards *Escherichia coli* as synonymous with fecal coliforms and does not give precise numerical values for colony counts. Baseline colony counts should be established for each supply system, and increases should be investigated. Most waterborne disease is related to fecal pollution of water sources; therefore, microbiologic testing of water needs to identify indicators of fecal pollution such as coliforms and *E. coli,* although the use of enterococci and *Clostridium perfringens* as surrogate markers is increasing. Coliforms must not be detected in 95% of samples when >50 samples are taken from the same sampling point during a 1-year period. Detecting *E. coli* in any one sample constitutes an infringement of the regulation. Recent U.K. legislation requires continuous monitoring of at-risk water treatment works for cryptosporidial oocysts (6). Supplying water containing >100 cryptosporidial oocysts per 100 L is a criminal offense; at least 1,000 L of water need to be filtered each 24 hours.

Address for correspondence: A. M. Emmerson, Department of Microbiology and Infectious Diseases and Nottingham Public Health Laboratory, University Hospital, Queen's Medical Centre, Nottingham NG7 2UH, United Kingdom; fax: 44-115-970-9233; e-mail: michael.emmerson@nottingham.ac.uk

### Private Water Supplies

Private water supplies may be used solely for domestic purposes (category 1) or on a larger scale to supply nursing homes, hospitals, and houses (category 2). Approximately 1% of the U.K. population obtains water from a well, borehole, or spring, which may not be treated. The quality of water from private supplies must comply with the requirements given in the Private Water Supplies Regulations 1991 (7). Category 1 supplies are further divided into classes A-F, depending on the amount of water and number of people supplied. Monitoring private supplies is problematic since water quality can change with the weather and smaller supplies are monitored infrequently (8).

Outbreaks of cryptosporidiosis traced to tap water from the main supply are uncommon but may affect large numbers of people and cause public alarm. A recent report highlights a new problem of *Cryptosporidium parvum* contamination of filtered borehole water causing confirmed cases in 345 persons (9). Borehole supplies have been traditionally regarded as relatively pure sources of water, so this outbreak has implications for future monitoring and treatment of drinking water extracted from boreholes.

### Water Storage and Distribution

Water should be stored safely in large, protected reservoirs and treated at the source, often by coarse filtration. Water should be distributed in a purpose-designed system, under pressure in a chlorinated form (e.g., 0.5 ppm free residual chlorine). Storage tanks should be protected from extraneous contamination, including by birds and vermin, and should be free from bacteria, particularly *E. coli.* Distribution systems should be controlled and free of "dead legs" (conduits that are capped off or rarely used) and spurs; joints and leaks should be repaired by qualified plumbers using defined materials. Uncontrolled water supplies are readily contaminated with coliform bacteria, environmental mycobacteria, *Legionella* spp., and filamentous fungi.

### Water as a Reservoir of Hospital Pathogens

While >40 *Legionella* spp. are known, most outbreaks of Legionnaires' disease are caused by *Legionella pneumophila* serotypes 1 and 6; 600 to 1,300 cases are reported each year in the United States, although these figures may represent underreporting (10). Legionellae are naturally distributed in aquatic environments, growing best at temperatures of 25°C to 42°C. Colonization is enhanced by water stagnation and sediment buildup as a result of alterations in the plumbing of the complex distribution systems often found in hospital hot-water systems. Cooling towers are often implicated in hospital and community outbreaks. Wet cooling towers (if used) and cooling water systems should be regularly maintained, cleaned, and disinfected. Cooling towers readily generate fine water droplets, as they operate by spraying water onto a packing material through which there is a countercurrent flow of air. How systems become seeded with *Legionella* is unclear, but these organisms can colonize certain types of water fittings, pipework, and materials. In practice, *Legionella* is found in many recirculating and hot-water systems with no associated clinical infection; in fact, the number of organisms that cause infection has not been determined reliably and varies with host susceptibility and species of *Legionella*. For these reasons, routine water sampling for *Legionella* is not advocated, but sampling may sometimes be appropriate to check the efficiency of the water treatment regimen.

Water systems should be designed to minimize colonization and multiplication of bacteria. Water should not be allowed to stagnate and should be circulated at temperatures below 20°C or above 60°C. Storage tanks and calorifiers should be regularly inspected, cleaned, and disinfected. In reported cases of Legionnaires' disease in which hot-water systems were implicated, contaminated water droplets were most commonly disseminated by showers and by taps with spray heads (faucet aerators). System design is all important in preventing buildup of *Legionella*; actions that lessen the risk for clinical cases include removing dead legs, avoiding washers and gaskets made of natural rubber (nutrient source), replacing heavily scaled faucets and showerheads, and avoiding shock absorbers and pipe materials not made of copper or plastic. Conditions that affect the proliferation of legionellae include sludge, scale, rust, algae, and organic particulates thought to provide nutrients for growth. Infection can be minimized by good engineering practices supplemented by heat, disinfectants, and biocides (11).

### Clinical Disease

A confirmed case of Legionnaires' disease is defined as clinical or radiologic evidence of pneumonia and a microbiologic diagnosis by culture of *L. pneumophila* from respiratory specimens, or a fourfold rise in serum antibody levels against *L. pneumophila* serogroups (often serogroups 1 and 6). Testing for *L. pneumophila* antigen in urine, which is rapid and convenient, is becoming the most common diagnostic method. Clinical cases have also occurred because of the inhalation of water droplets containing the blue-white fluorescent group of legionellae, e.g., *L. gormanii* and *L. bozemanii*. Care must be taken with the indirect immunofluorescent antibody test to absorb any cross-reactions from *Campylobacter*. Immunocompromised patients, e.g., transplant or dialysis patients or those on cytotoxic therapy, are at higher risk for infection with *Legionella*.

### Legionellosis: Control by Disinfection

Ideally, hospital water systems should be free of legionellae, but it is exceptional for a water supply to be entirely free of aquatic organisms. Provided that water is derived from the public mains and its quality is preserved in the storage and distribution system by correct design, installation, and maintenance, it can be regarded as being microbiologically acceptable for use without further treatment. However, if the appropriate detection systems are in place to culture and detect nonculturable organisms, it is likely that legionellae will be found in distribution systems (12). Marrie et al. demonstrated that a water system may be contaminated without clinical consequence (13), although risk should be assessed. If regular prospective surveillance and environmental cultures are undertaken and low levels ($<10^2$ per L) of legionellae are found, no action is necessary; counts of $10^2$ to $10^3$ on successive samples warrant a review of control procedures.

### Heat

If storing water at 60°C is not practical or acceptable or the calorifier is not in use for 1 week or more, raising the temperature of the calorifier water to 70°C to 75°C for 1 hour will kill legionellae. However, this technique may not be effective if the temperature of water at the bottom of the calorifier does not reach 70°C.

### Chlorination

Hot-water systems can be disinfected by chlorinating the water in the header tank (20 ppm to 50 ppm, superchlorination), allowing the water to flow to all parts of the system, and then allowing it to stand for at least 4 hours while not in use. The system should then be completely and thoroughly flushed before use. Cooling towers and cooling water systems can be chlorinated with 5 ppm for several hours before flushing. Water in a cleaned system can then be dosed to give a circulating level of free residual chlorine of approximately 1 ppm, although this may increase corrosion.

### Biocides

Some biocides are effective against legionellae if used in sufficient concentrations for a sufficient time. Alternating high-level biocide treatment with chlorination and shock-dosing the water system are likely to be more effective than continuous low-level dosing with a single biocide. Strategies for preventing Legionnaires' disease (14) and guides to minimizing the risk are available (15).

### Other Disinfection Methods

Copper-silver ionization can be used to control legionellae in hospital hot-water recirculating systems (16). This method electrically generates copper and silver ions, which bind to the bacterial cell wall, causing cell-wall disruption and lysis. Other methods for disinfecting drinking water include ozonation, chlorine dioxide, and irradiation by UV light.

### *Legionella* spp. and Free-Living Protozoa

Legionellae thrive in stagnant water at ambient temperatures and may survive chlorination by residing in sludge and scale or inside certain protozoa, e.g., *Acanthamoeba*, *Hartmannella*, and *Tetrahymena* spp. While legionellae and most protozoan trophozoites are inactivated by 1 ppm to 2 ppm of free residual chlorine, some protozoan cysts can resist 50 ppm chlorine; intracellular legionellae may be more resistant than the planktonic forms (17).

### Rinse Water as a Source of Hospital Pathogens

Automatic washer-disinfector systems are widely used for decontaminating flexible fiberoptic endoscopes. These expensive scopes may be cleaned and decontaminated manually in individual diagnostic units or in centralized endoscopy-decontamination units. The main water supply may contain environmental microorganisms, such as mycobacteria, legionellae, and aerobic gram-negative bacilli, which may recontaminate the endoscope during rinsing. Pseudoepidemics of *L. pneumophila* serogroup 6 associated with contaminated bronchoscopes have been reported (18), as has the transmission of highly drug-resistant *Mycobacterium tuberculosis* caused by inadequate cleaning and disinfection of a bronchoscope (19). Hospital water supplies can readily become contaminated with environmental mycobacteria, e.g., *M. xenopi, M. abscessus, M. fortuitum*, and *M. chelonae*; if decontamination units do not have filters (0.2 µm) fitted to the water supply, rinse water may become contaminated. Water filters need to be fitted and maintained, but even this filtration system does not guarantee bacteria-free water (20). Environmental mycobacteria such as *M. chelonae* can resist temperatures of 45°C and some disinfectants such as 2% alkaline glutaraldehyde. Washer-disinfectors should be installed and maintained according to manufacturer's recommendations. Management policies should emphasize regular cleaning and maintenance (21). Use of contaminated or hard water should be avoided to lessen formation of biofilm and buildup of lime scale. Use of poor-quality water also should be avoided, and the supply to the washer-disinfector should be pretreated with heat and filtration and other processes such as UV irradiation and reverse osmosis. Additional chlorination of the water also should be considered, as should a final endoscope rinse with sterile water (22).

### Immersion in Water

#### Hydrotherapy Pools: Preventing Infection

The physical structure of hydrotherapy pools, their high water and air temperatures, and intermittently intensive use by diverse groups of patients and staff produce potentially hazardous conditions (23). Hydrotherapy has become popular, and many district hospitals have installed suitable pools. Each pool should be a self-contained part of the hospital physiotherapy facilities with a senior physiotherapist responsible for overall daily management. The pool should be designed to allow water to circulate through a filter and for the addition of a suitable disinfectant (often hypochlorite) in appropriate amounts with a mechanism for adjusting the pH (appropriate range 7.2 to 7.8). Pools should be cleaned regularly, have some water replaced weekly, and be emptied annually. Additional measures should be implemented if users release unformed stool into the pool, and strict adherence to the rules of cleanliness and hygiene both in and out of the pool should be enforced. Physiotherapists, microbiologists, and engineers should have effective working relationships. Management programs should be established, and careful records should be kept. Despite careful control of water quality, users will suffer from pool-related skin, ear, chest, and gastrointestinal infections from time to time. Numerous microorganisms have been implicated in these infections, including *Pseudomonas aeruginosa*, *Legionella* spp., adenoviruses, and enteroviruses. Legionnaires' disease has been associated with whirlpool spas, where agitation and aeration of the water enable bacteria to be inhaled (24). (The terms spa pool, spa bath, whirlpool, and hot tub are sometimes used interchangeably [25]). More recently, a cluster of gastrointestinal illnesses, including one case of hemolytic uremic syndrome and one culture-confirmed *E. coli* O157:H7 infection, was attributed to a poorly maintained swimming pool (26). Frequently, immersion of hospitalized patients contaminates the tub environment, including the tub water, drains, agitators, floors, and walls.

### Water Births: Minimizing Infection

Water births, pioneered in the 1960s, are increasingly being used. The perceived infection problem is that the birthing-pool water becomes contaminated with amniotic fluid, blood, and fecal material, all of which contain large quantities of maternal bacteria and viruses. Risks include bloodborne viruses, e.g., hepatitis B and C, HIV-1, and HIV-2, and fecal-orally transmitted viruses, e.g., the enteroviruses and adenoviruses (27). Many of these concerns may be unfounded, and calls for maternal testing for HIV have not been supported. A more reasonable approach is to ensure that infection control policies for water births include instructions for pool maintenance and decontamination, use of universal precautions, and use of personal protective equipment for staff (28). Postnatal surveillance of mothers and babies should be conducted to define infection rates.

### Washing or Rinsing in Water

#### Burns Units: Part of Irrigation Therapy

Kolmos et al. reported five patients with extensive deep burns in whom *P. aeruginosa* serogroup 0-7 septicemia developed shortly after hospital admission (29). Routine microbiologic monitoring of such patients is not required, provided the water quality is secured and the irrigation tubing is decontaminated between uses.

#### Bathing Infants: Basic Hygiene and Appearance

At birth, infants are often diffusely covered in vernix, amniotic fluid, and blood. Even though bathing them to remove unsightly body fluids is very tempting, total body immersion for preterm babies is not recommended. The skin of a newborn is ideal for absorbing unwanted microorganisms. In a report by Verweij et al., contaminated water was used to wash preterm infants, leading to the colonization of four infants and death of a fifth from *Stenotrophomonas maltophilia* (30). The outbreak was controlled by reenforcing hand disinfection, limiting use of tap water for handwashing, and using sterile water to wash the preterm babies. For cosmetic reasons, washing can be restricted initially to the head and neck.

### Miscellaneous Waterborne Outbreaks

Water baths used to warm up dialysis fluids (31), fresh-frozen plasma, and albumin (32) have been implicated as the source of infection by *Acinetobacter calcoaceticus* var *anitratum* and *P. aeruginosa*. Molecular methods such as pulsed-field gel electrophoresis or random amplification of polymorphic DNA can confirm the relatedness of some of these complex aerobic gram-negative bacilli. Removing the contaminated water baths ends the outbreaks.

Holy water is a potential source of cross-infection with various coliform bacteria, including *A. baumanii* and *Aeromonas hydrophila* (33). Patients with widespread burns and other debilitating skin lesions are at risk. Sterile holy water is one solution to this concern.

A number of pseudooutbreaks have been reported that implicate contaminated ice machines. Coliforms and environmental mycobacteria such as *M. gordonae* are frequently found in the water source (34). Pseudoinfection by *M. gordonae* and others can be prevented by adequate machine maintenance.

An outbreak of group A hemolytic streptococcal puerperal sepsis was traced to the communal use of bidets (35). Decontamination of the water spray nozzle and drain was necessary to control the outbreak. Routine cleaning might have prevented its occurrence.

### Acknowledgments

The author thanks Karen Kennedy for typing the manuscript and D. Greenwood and John Lee for their thoughtful review.

Dr. Emmerson is professor of clinical microbiology and head of the Department of Microbiology and Infectious Diseases at the University of Nottingham. His main research interests include the control of hospital-acquired infection, sterilization of heat-sensitive and heat-resistant instruments, and quality standards and use of hospital-associated equipment.

### References

1. Standing Committee of Analysts (1994). The microbiology of water (1994). Part I. Drinking water. Methods for the examination of waters and associated materials. Reports on Public Health and Medical Subjects No. 71. London: Her Majesty's Stationery Office; 1994.
2. The Water Supply (Water Quality) Regulations 1989 Statutory Instruments 1989 No. 1147. London: Her Majesty's Stationery Office; 1989.
3. Lewis MJ. Water fit to drink? Microbial standards for drinking water. Rev Med Microbiol 1991:2:1–6.
4. Council Directive of 15th July 1980—relating to the quality of water intended for human consumption (80/778/EEC). Official Journal of European Communities 1980;L229:11–28.
5. European Union. Council Directive 98/83/EC on the quality of water intended for human consumption. Official Journal of European Communities 1998;L330:32–54.
6. The Private Water Supply (Water Quality) (Amendment) Regulations 1999. Statutory Instrument 1999 No. 1524. London: Her Majesty's Stationery Office; 1999.
7. The Private Water Supplies Regulations 1991. Statutory Instrument 1991. No. 2790. London: Stationery Office; 1991.
8. Barrell RAE, Hunter PR, Nichols G. Microbiological standards for water and their relationship to health risk. Commun Dis Public Health 2000;3:8-13.
9. Willocks L, Crampin A, Milne L, Sang C, Susman M, Gair R, et al. A large outbreak of *Cryptosporidiosis* associated with public water from a deep chalk borehole. Commun Dis Public Health 1998;1:239-43.
10. Goetz A, Yu V. Nosocomial *Legionella* infection. In: Mayhall C, editor. Hospital epidemiology and infection control. Baltimore: William and Wilkins; 1996.
11. Health Technical Memorandum 2040. The control of *Legionellae* in healthcare premises—a code of practice. London: Her Majesty's Stationery Office; 1993. (ISBN 011321 6807).
12. Goetz AM, Stout JE, Jacobs SL, Fisher MA, Ponzer RE, Drenning S, et al. Nosocomial *Legionnaires*' disease discovered in community hospitals following cultures of the water system: seek and ye shall find. Am J Infect Control 1998;26:8-11.
13. Marrie T, MacDonald S, Clarke F, Haldane D. Nosocomial *Legionnaires*' disease: lesson from a four-year prospective study. Am J Infect Control 1991:19:79-85.
14. Ruef C. Nosocomial *Legionnaires*' disease. Strategies for prevention. J Microbiol Methods 1998;33:81-91.
15. Freije MR. *Legionellae* control in health care facilities–a guide to minimizing risks. Indianapolis: HC Information Resources Inc.; 1996.
16. Stout JE, Lin YSE, Goetz AM, Muder RR. Controlling *Legionella* in hospital water systems: experience with superheat and flush method and copper–silver ionization. Infect Control Hosp Epidemiol 1998;19:911-4.
17. Patterson WJ, Hay J, Seal DV, McLuckie JC. Colonization of transplant unit water supplies with *Legionella* and protozoa: precautions required to reduce the risk of legionellosis. J Hosp Infect 1997;37:3-17.
18. Mitchell DH, Hicks LJ, Chiew R, Montanaro JC, Chen SC. Pseudo epidemic of *Legionella pneumophila* serogroup 6 associated with contaminated bronchoscopes. J Hosp Infect 1997:37:19-23.
19. Agerton T, Valway S, Gore B, Pozsik C, Plikaytis B, Woodley C, et al. Transmission of a highly drug-resistant strain (strain WI) of *Mycobacterium tuberculosis*. JAMA 1997;278:1073-7.
20. Cooke RPD, Whymant-Morris A, Umasankar RS, Goddard SU. Bacteria-free water for automatic washer-disinfectors: an impossible dream. J Hosp Infect 1998;38:63-5.
21. Hospital Technical Memorandum 2030. Washer disinfectors. London: Her Majesty's Stationery Office; 1995.
22. Marrie TJ, Haldane D, MacDonald S, Clarke F, Fanning C, Le Fort-Jost S, et al. Control of endemic Legionnaires' disease by using sterile potable water for high risk patients. Epidemiol Infect 1991;107:591-605.
23. Public Health Laboratory Service. Hygiene for hydrotherapy pools. 2nd ed. London: PHLS; 1999. (ISBN 090 1144 460).
24. Jernigan B, Hoffman J, Cetron M. Outbreak of legionnaires' disease among cruise-ship passengers exposed to a contaminated whirlpool spa. Lancet 1996;347:494-9.
25. Dadswell J. Managing swimming, spa and pools to prevent infection. Commun Dis Rep CDR Rev 1996;6:R37-R40.
26. Friedman MS, Roels T, Koehler JE, Feldmann L, Bibb WF, Blake P. *Escherichia coli* O157:H7 outbreak associated with an improperly chlorinated swimming pool. Clin Infect Dis 1999;29:298-303.
27. Ridgway GL, Tedder RS. Birthing pools and infection control. Lancet 1996;347:1051-2.
28. Kingsley A, Hutter S, Green N, Spiers G. Waterbirths; regional audit of infection control practices. J Hosp Infect 1999;41:155-7.
29. Kolmos HJ, Thuesen B, Nielsen SV, Lohmann M, Kristoffersen K, Rosdahl VT. Outbreak of infection in a burns unit due to *Pseudomonas aeruginosa* originating from contaminated tubing used for irrigation of patients. J Hosp Infect 1993;24:11-21.
30. Verweij PE, Meis JFGM, Christmann V, Van der Bor M, Melchers WJG, Hilderink BGM, et al. Nosocomial outbreak of colonization and infection with *Stenotrophomonas maltophilia* in pre-term infants associated with contaminated tap water. Epidemiol Infect 1998;120:251-6.

31. Ashline V, Stevens A, Carter MJ. Nosocomial peritonitis related to contaminated dialysate warming water. Am J Infect Control 1981;9:50-2.
32. Muyldermans G, De Smet F, Pierand S, Steenssmens L, Stevens D, Bougatef F, et al. Neonotal infections with *Pseudomonas aeruginosa* associated with a water-bath used to thaw fresh frozen plasma. J Hosp Infect 1998;39:309-14.
33. Rees JC, Alten KD. Holy water—a risk for hospital-acquired infection. J Hosp Infect 1996;32:51-5.
34. Panwalker AP, Fuhse E. Nosocomial *Mycobacterium gordonae* pseudo infection from contaminated ice machines. Infect Control 1986;7:67-70.
35. Gordon G, Dale BAS, Lochhead D. An outbreak of group A haemolytic streptococcal puerperal sepsis spread by the communal use of bidets. Br J Obstet Gynecol 1994;101:447-8.
